# Revisiting Preclinical Observations of Several Histamine H3 Receptor Antagonists/Inverse Agonists in Cognitive Impairment, Anxiety, Depression, and Sleep–Wake Cycle Disorder

**DOI:** 10.3389/fphar.2022.861094

**Published:** 2022-06-01

**Authors:** Mera Alhusaini, Nermin Eissa, Ali K. Saad, Rami Beiram, Bassem Sadek

**Affiliations:** ^1^ Department of Pharmacology and Therapeutics, College of Medicine and Health Sciences, United Arab Emirates University, Al Ain, United Arab Emirates; ^2^ Zayed Center for Health Sciences, United Arab Emirates University, Al Ain, United Arab Emirates; ^3^ Department of Biomedical Sciences, College of Health Sciences, Abu Dhabi University, Abu Dhabi, United Arab Emirates

**Keywords:** neurotransmitters, histaminergic system, neurological disorders, H3R, antagonists, H3R inverse agonists

## Abstract

A relationship appears to exist between dysfunction of brain histamine (HA) and various neuropsychiatric brain disorders. The possible involvement of brain HA in neuropathology has gained attention recently, and its role in many (patho)physiological brain functions including memory, cognition, and sleep–wake cycle paved the way for further research on the etiology of several brain disorders. Histamine H3 receptor (H3R) evidenced in the brains of rodents and humans remains of special interest, given its unique position as a pre- and postsynaptic receptor, controlling the synthesis and release of HA as well as different other neurotransmitters in different brain regions, respectively. Despite several disappointing outcomes for several H3R antagonists/inverse agonists in clinical studies addressing their effectiveness in Alzheimer’s disease (AD), Parkinson’s disease (PD), and schizophrenia (SCH), numerous H3R antagonists/inverse agonists showed great potentials in modulating memory and cognition, mood, and sleep–wake cycle, thus suggesting its potential role in neurocognitive and neurodegenerative diseases such as AD, PD, SCH, narcolepsy, and major depression in preclinical rodent models. In this review, we present preclinical applications of selected H3R antagonists/inverse agonists and their pharmacological effects on cognitive impairment, anxiety, depression, and sleep–wake cycle disorders. Collectively, the current review highlights the behavioral impact of developments of H3R antagonists/inverse agonists, aiming to further encourage researchers in the preclinical drug development field to profile the potential therapeutic role of novel antagonists/inverse agonists targeting histamine H3Rs.

## 1 Introduction

Neuropsychiatric disorders are complex conditions characterized by cognitive deficits, mental health symptoms, and features with poorly defined neurobiological bases. Major neurodegenerative disorders including Alzheimer’s disease (AD) and Parkinson’s disease (PD), and neuropsychiatric diseases such as schizophrenia (SCH) and depression are recognized as chronic diseases beginning early in life and affecting patients across all age groups, with underlying biological mechanisms largely unknown (Kessler et al., 2007; Shan et al., 2015; Bray and O'Donovan, 2019; Carthy and Ellender, 2021). These conditions are a major public health challenge with high prevalence, diminishing quality of life for millions of patients and their caregivers (Kessler et al., 2005; Gooch et al., 2017). Several pharmacological agents for neuropsychiatric disorders were developed and approved. However, their mechanisms are still incompletely understood; hence, further drug discoveries remain an area of active research. Alterations in the neurotransmission can lead to many pathological changes that occur in brain disorders, demonstrating the importance to illuminate the pathogenesis of neuropsychiatric disorders and to develop new potential pharmacological agents. Accumulating evidence suggests the interplay of brain neurotransmitters in several neurological disorders. Evidence exists for both cholinergic and glutamatergic involvement in the etiology of AD. In addition, the potential synergy between cholinesterase inhibitors and the memantine, an *N*-methyl-d-aspartate (NMDA) receptor antagonist, has been addressed in the improvement of neurologic abnormalities associated with AD (Francis, 2005). Also, brain dopamine (DA) and histamine (HA) have influences on behavior in brain disorders including AD, SCH, anxiety, and narcolepsy, all of which show overlap in their features and symptoms (DiCarlo et al., 2019). Brain histaminergic signaling has frequently been reported to influence several neuropsychiatric disorders. Moreover, the brain histaminergic system was found to display a critical role in cognition and sleep, and also in several neuropsychiatric disorders including AD, PD, SCH, and Tourette syndrome (Wright et al., 2017). The significant involvement of brain DA and HA in the latter brain disorders substantiates these neurotransmitter systems as being a significant area in studying the etiology of such brain diseases.

### 1.2 Histaminergic Brain Signaling

Brain HA exerts its effects through the activation of four G-protein-coupled receptors, namely, HA receptors (HRs) H1R, H2R, H3R, and H4R, which are often distributed in the brain, smooth muscles, gastric cells, as well as the bone marrow with distinct subsequent physiological functions. Many of H1R functions contribute to allergic responses (Kapalka, 2010), while H2R induces airway mucus production, vascular permeability, and gastric acid secretion (Smit et al., 1996). H3Rs mainly contribute in numerous functions of the central nervous system (CNS) (Lovenberg et al., 1999). As for H4R, it mediates inflammatory disorders, allergy, autoimmune disease, and cancer, as briefly discussed by Zampeli and Tiligada, (2009).

The functions and impacts of H3Rs have raised interest in the fields of brain and CNS research. It is mainly expressed in the cerebral cortex, thalamus, and ventromedial nucleus of the hypothalamus. H3R is distinguished from other subtypes by being a presynaptic inhibitory H3-autoreceptor that controls the synthesis and release of HA in a negative feedback mechanism, and as a presynaptic H3-heteroreceptor localized on non-histaminergic neurons modulating the release of numerous other neurotransmitters including DA, serotonin (5-HT), noradrenaline (NA), GABA, and acetylcholine (ACh) (Jutel et al., 2009). The involvement of brain histaminergic system in neuropsychiatric disorders has gained attention in the research of disease pathophysiology and has been accounted responsible for multiple abnormalities in the brain. Significant efforts have recently been made in the development of new agents that specifically target the histamine H3Rs for the treatment of AD, PD, SCH, depression, and other cognitive disorders (Panula and Nuutinen, 2013; Shan et al., 2015). H1R, H2R, and H3R are expressed in high densities in brain regions involved in cognition, highlighting the significant role of the brain histaminergic system in cognitive functions. Schneider et al. (1997) reported a decrease in histidine decarboxylase (HDC) activity that was found to be parallel to the decrease in the activity of choline acetyltransferase in patients with AD, although no significant decrease in brain HA levels had been observed (Schneider et al., 1997). On the other hand, another study stated loss of histaminergic neurons in patients with AD accompanied with decreased levels of brain HA metabolite in cerebrospinal fluid which indicates impaired HA activity (Naddafi and Mirshafiey, 2013). Nevertheless, different abnormalities in the brain histaminergic system have also been reported in SCH patients. Accordingly, reduced expression of H1R in the frontal cortex of chronic SCH patients was reported, along with reduced H1R binding in the frontal and prefrontal cortices and the cingulate gyrus (Iwabuchi et al., 2005). H3Rs possess a unique role in controlling the release of HA in addition to other neurotransmitters affecting various complex brain functions. Therefore, improvement of cognitive functions can be achieved via the modulation of H3Rs, evoking the increase in neuronal HA release, thus allowing the neurotransmitter to modulate cognitive functions either directly by interacting with postsynaptically located H1- and H2Rs or indirectly through the modulation of the cholinergic, dopaminergic, and GABAergic neurotransmissions (Haas and Panula, 2003; Sadek et al., 2016a; Sadek and Stark, 2016). This role renders agents such as H3R antagonists/inverse agonists as attractive therapeutic targets in CNS diseases. Moreover, modulation of the sleep–wake cycle was also described to be mediated by HA levels in the brain (Brown et al., 2001; Haas et al., 2008; Fabara et al., 2021).

## 2 Neurological Disorders and Alterations in Neurotransmitters

### 2.1 Alzheimer’s Disease

AD is a progressive neurodegenerative disorder affecting wide areas of the cerebral cortex and the hippocampus. Also, AD is multifactorial and associated with many different genetic risk loci, with the apolipoprotein E ε4 allele being a major genetic risk factor for late-onset AD (Nikolac Perkovic and Pivac, 2019). The primary event of AD pathogenesis is the accumulation of the insoluble form of the protein amyloid-β (Aβ) extracellularly (Masters et al., 2015). A study by Näslund *et al.* presented compelling evidence that the presence of these protein fragments in areas involved in memory and cognition is considered an early hallmark that anticipates neurological impairment and development of proteinaceous lesions (Näslund et al., 2000). Hyperphosphorylated microtubule protein, namely tau, often accumulated in neurofibrillary tangles of neurons is another protein that may be involved in the pathogenesis of AD. Several studies imply that tau formation is a result of an abnormality in Aβ production and clearance (Hardy and Selkoe, 2002). Recently, a study suggested that the relation between Aβ and tau protein is not only one of co-existence, rather a pathogenic interaction that drives the progression of the disease (Busche and Hyman, 2020). The main clinical manifestation of AD is cognitive decline that progresses throughout the course of the disease. Jessen *et al.* primarily described AD as a progressive decline in objective or subjective cognitive capacity, known as stage 1 that further worsens to developing severe dementia which is clinically referred to as stage 6 (Jessen et al., 2020).

Moreover, loss of neurons and the presence of neurofibrillary tangles have been reported in the tuberomammillary nucleus of the hypothalamus of AD patients’ brain (Nakamura et al., 1993), which is an area of the brain where neuronal HA cell bodies are localized (Brown et al., 2001). Furthermore, alterations in the neuronal histaminergic system have been recognized to contribute to the cognitive impairments displayed by AD patients (Passani and Blandina, 2011; Sadek et al., 2016a; Sadek and Stark, 2016; Zlomuzica et al., 2016). Accordingly, several lines of evidence showed that AD patients exhibit alterations in HA brain levels, decreased expression of H1Rs in the frontal and temporal cortices, and degeneration of histaminergic neurons in the tuberomammillary nucleus (Airaksinen et al., 1991; Zlomuzica et al., 2016). The reduced amount of H1R binding reported in the frontal and temporal areas of AD patients has been correlated with the severity of their cognitive symptoms, suggesting that a decrease in H1R expression contributes to the observed cognitive deficits in AD patients due to changes in the histaminergic neurotransmission (Higuchi et al., 2000).

### 2.2 Parkinson’s Disease

Being the second most common neurodegenerative disease, PD gained great attention since James Parkinson’s assay over 200 hundred years ago (Mhyre et al., 2012). The etiology of PD remains currently unknown; however, the genetic background of the disease is nowadays well established, as the majority of PD cases are sporadic, probably caused by a combination of genetic and environmental risk factors (Kalinderi, et al., 2016). It is characterized by two neuronal features that are necessary for the definitive diagnosis of PD: first, a dopaminergic neuronal loss in the areas of the substantia nigra and, second, accumulation of intracellular protein (α-synuclein) (Poewe et al., 2017). A recent review of post-mortem brain studies for PD patients concluded that there might be an increase in local HA release in regions such as the substantia nigra, implicating the role of histaminergic system in the pathophysiology of PD (Shan et al., 2015). In addition to research on postmortem samples, preclinical animal models were and are still used to study the pathogenesis of PD. The injection of irreversible inhibitor of HDC, namely, α-fluoromethylhistidine (α-FMH), in 6-hydroxydopamine (6-OHDA)-lesioned rats, which is a classic PD model, showed a significant decrease in the rotation behavior induced by apomorphine on day 14 post-lesion. Additionally, it prevented the loss of tyrosine hydroxylase (a marker for dopaminergic neurons) expressing cells (Liu C. et al., 2007). Moreover, HDC, H1R/H2R antagonists, and H3R agonists were described to display ameliorating effects in the apomorphine-induced turning behavior in the 6-OHDA-lesioned rats (Liu C. et al., 2007). Interestingly, H3R antagonist/inverse agonists such as thioperamide were found to increase the brain level of HA and to alleviate apomorphine-induced behavioral responses in rats, and could also rescue the memory impairments in the mouse model of PD (Nowak et al., 2009; Masini et al., 2017). The latter observations signify the important role of dysregulated histaminergic system in the pathophysiology of PD.

### 2.3 Schizophrenia

SCH is a crippling disease that affects multiple functions in the brain such as emotions and cognition. Despite having a relatively low prevalence, SCH is considered a clinical challenge due to its complexity, and a socioeconomic burden as a result of its early onset during late adolescence or early adulthood (Charlson et al., 2018). Dysregulation of the brain histaminergic system and abnormal brain HA neurotransmission have been reported to be associated with several features of SCH. In clinical studies, elevated levels of a major HA metabolite, namely, *N* ^τ^-methylhistamine, suggested a greater release and turnover of brain HA in the cerebrospinal fluid of SCH patients (Ito, 2004). Moreover, H1R expression in cholinergic neurons in the basal forebrain was reported to be lower in SCH patients, and the deletion of H1Rs in these neurons in mice exhibited sensorimotor gating impairments, social deficits, and anhedonia-like behaviors (Cheng et al., 2021). In addition, several H2R antagonists have been shown to reduce both the positive and negative symptoms of SCH (Meskanen et al., 2013; Mehta and Ram, 2014). Furthermore, upregulation of H3Rs expression was observed in the prefrontal cortex of people with SCH (Jin et al., 2009), with previous preliminary clinical studies demonstrating improvement in cognitive impairment by H3R antagonists (Bardgett et al., 2010; Brown et al., 2013). The impairment of the cholinergic system is also reported to be implicated in SCH. Some researchers indicated that cholinergic, basically the muscarinic, systems play a vital role in the pathogenesis of SCH after the onset (Raedler et al., 2007). Several pieces of evidence in postmortem studies supported the decrease in the levels of muscarinic receptors in large areas of the brain in SCH patients (Mancama et al., 2003; Katerina et al., 2004). Another study of brains of SCH patients reported a marked decrease in muscarinic receptor expression in the hippocampus and prefrontal cortex that are projected from brainstem cholinergic neurons (Crook et al., 2000). Also, replicated findings suggested that muscarinic receptor reduction in SCH may be disease specific, as cholinergic dysfunction plays a crucial role in positive symptoms, negative symptoms, cognitive impairments, and autonomic and motor functions in patients with SCH (Raedler et al., 2003). Moreover, when the ACh level declines critically, the cholinergic receptors, mainly muscarinic receptors, are widely disrupted in the case of SCH, leading to various symptoms of pathophysiology in SCH (Tani et al., 2015).

Regardless of the aforementioned observations for the capability of numerous H3R antagonists/inverse agonists to mitigate schizophrenic features in experimental rodents, clinical trials revealed negative or, at most, moderate outcomes. Accordingly, and in a clinical study, the brain penetrant and highly potent H3R antagonist/inverse agonist GSK239512 was generally well tolerated, but failed to provide overall beneficial effects on cognitive impairments associated with SCH (Jarskog et al., 2015). Moreover, and in another clinical study, the highly potent H3R antagonist/inverse agonist ABT-288 showed an increased incidence of psychosis-related and sleep-related adverse events. The study also concluded that medication with ABT-288 resulted in cognitive improvement in clinically stable adults diagnosed with SCH (Haig et al., 2014a). In addition, the H3R antagonist/inverse agonist MK-0249 (10 mg once daily) failed to be superior to placebo in the treatment of cognitive impairment in patients with SCH and following medications for 4 weeks (Egan et al., 2013).

## 3 Neurological Manifestations and Application of Several H3R Antagonists/Inverse Agonists in Attenuating Neurological Symptoms in Preclinical Models

### 3.1 Memory Impairment

**FIGURE 1:**
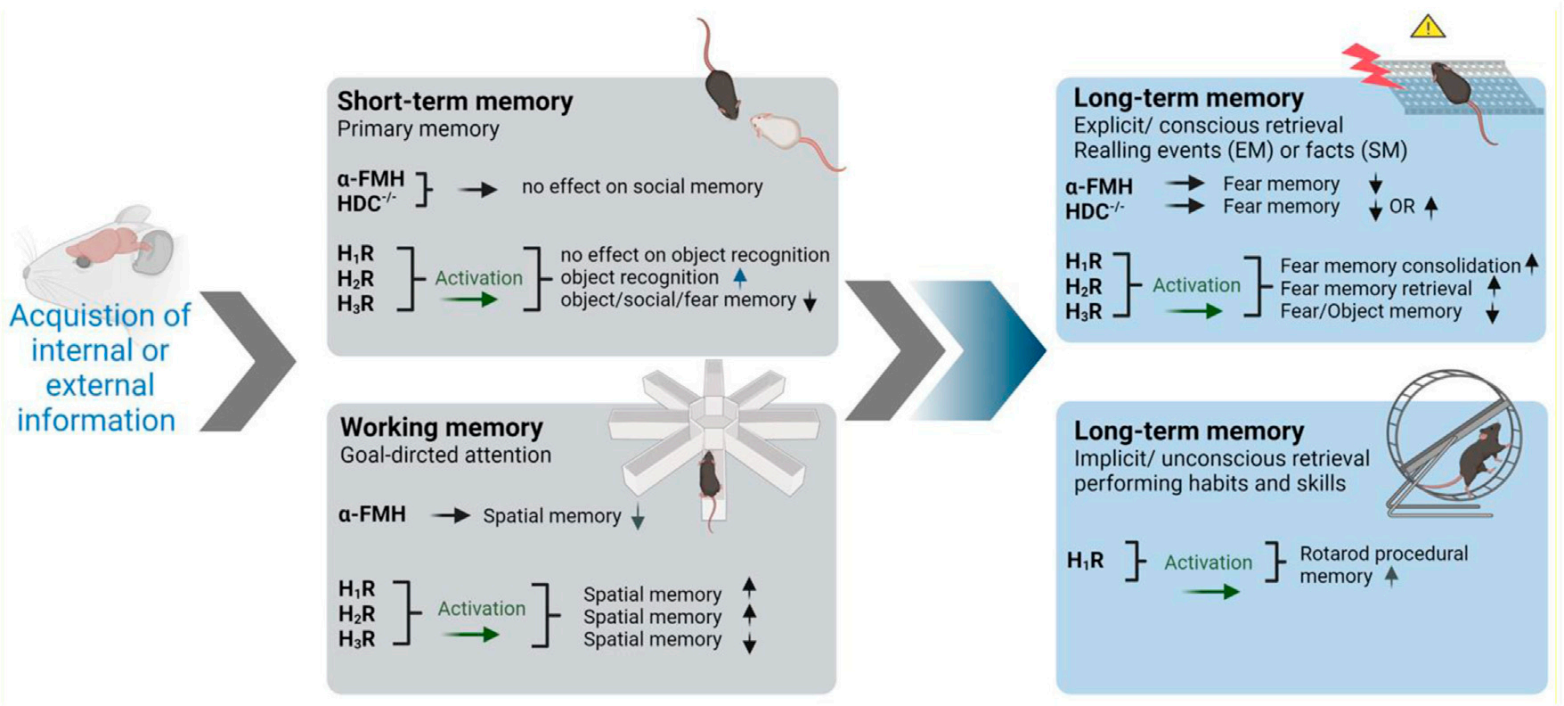
Involvement of brain histamine in various types and stages of memory in preclinical studies. Symbolic photos were used to represent the most utilized behavioral task in each category. α-FMH, α-fluoromethylhistamine; HDC-/-, histidine decarboxylase homozygous knockout mice; EM, episodic memory; SM, semantic memory. The arrows pointing up indicate improved memory. The arrows pointing down indicate decrease in memory performance. Graphics were constructed utilizing BioRender software program and were also licensed for publication.

Impairment of memory is one of many neurological manifestations expressed in some neuropathies such as AD and PD. However, the affected type of memory differs between the pathologies. In humans, there are two main classifications of memory depending on the time interval in which the brain stores information: short-term memory (working memory) and long-term memory. In general, memory formation passes through three main consecutive steps: acquisition of sensory and internal information, consolidation of such information, and finally retrieval after short or long duration (Cowan 2008). Interestingly, the first suggestion that brain HA plays a significant role in memory consolidation was from de Almeida and Izquierdo group who observed that the immediate post-training i.c.v. administration of 1–10 ng HA was found to improve the retention of one-trial inhibitory avoidance in rats (de Almeida and Izquierdo, 1986). The later significant observations rely on hippocampal CA1 long-term potentiation, as ascertained by measurement of cellular biochemical changes (Izquierdo et al., 2006) and shortly after by electrophysiological observations (Whitlock et al., 2006; Izquierdo et al., 2016), and were found to be strongly modulated by the basolateral amygdala (McGaugh, 2004; McGaugh, 2015). Moreover, a preclinical significant finding revealed that the trace is stored in parallel in hippocampal CA1 and in basolateral amygdala (Izquierdo et al., 1992; Fabbri et al., 2016; Izquierdo et al., 2016). In the following sections, available data on histamine neurotransmission contribution to different types and phases of memory will be discussed and are schematically summarized in Figure 1.

#### 3.1.1 Short-Term Memory

New sensory or internal information will be encoded initially through primary, short-term processing (Cowan, 2008). Different brain circuits and regions convey various short-term memories. Prefrontal cortex maintains short-term memory by selecting relevant sensory information and ignoring irrelevant information to aid performance in tasks such as object recognition (Courtney, 2010). Additionally, other brain structures including basal ganglia, perceptual, and motor cortices are involved in STM (Courtney, 2010). Several studies indicate that distinct neuronal circuits are recruited in the processing of visuospatial (occipitoparietal cortex), auditory (temporal cortex), or phenological (inferior parietal cortex) STM systems (Vallar, 2017). Furthermore, temporary changes in synapses through neurotransmitters, including HA, and their downstream signaling pathways were found to be involved in STM (Mallakin, 2020). Interestingly, i.c.v. administration of HA and its precursor, l-histidine, improved social recognition memory assessed in experimental rodents (Prast et al., 1996). In the same study, H3R antagonist thioperamide provided the same behavioral results. However, a combination of genetic and pharmacological approaches comprehended the involvement and the requirement of the brain histaminergic system in social recognition learning using the social discrimination paradigm in mice. Hereafter, pharmacological or genetic disruption of the histaminergic system, through α-FMH or HDC^-/-^, respectively, failed to disturb short-term recognition memory or fear memory in the inhibitory avoidance paradigm in mice (Rani et al., 2021). Accordingly, VUF16839, an agonist targeting H3Rs, was found to impair performance of tested animals in short-term social recognition in normal and, surprisingly, in HA-depleted mice (Rani et al., 2021), and this impairment was reversed by donepezil, suggesting that VUF16839 activated H3-heteroreceptors that control the release of ACh rather than HA (Rani et al., 2021). Furthermore, H3R antagonists/inverse agonists thioperamide and ciproxifan were described to ameliorate spatial memory deficits (Komater et al., 2005). In addition, the H3R antagonists/inverse agonists E159 and E177 were found to enhance short-term object recognition comparable to the reference drug donepezil, clinically used acetylcholinesterase inhibitor. Moreover, and in the same study, systemic co-administration of brain penetrant H2R antagonist zolantidine nullified the beneficial effects provided by E159 on memory deficits (Alachkar et al., 2017; Alachkar et al., 2019). Contrarily, DL77, another H3R antagonist/inverse agonist, failed to improve short-term memory but enhanced long-term memory in the same behavioral tasks (Eissa et al., 2018). Also, and in another preclinical study, H3R antagonist/inverse agonist CEP-26401 was found to improve social recognition memory in tested animals (Raddatz et al., 2012).

#### 3.1.2 Working Memory and Histaminergic Neurotransmission

Although many theories and classifications exist, working memory can be viewed as short-term memory combined with goal-directed attention (Cowan, 2008). Attention among other executive brain functions is regulated by the prefrontal cortex, which interacts with other brain regions in maintaining working memory (D'Esposito, 2007). In regard to the role of brain histaminergic neurotransmission in working memory, depletion of brain HA following i.c.v. administration of the HDC inhibitor α-FMH or blockade of H1Rs but not H2Rs was found to disturb cognitive functions in experimental animals in eight-arm radial maze. Interestingly, and in agreement with the latter observations, H1R antagonist but not H2R antagonist was found to impair working memory, while H3R antagonist/inverse agonist clobenpropit was reported to improve cognitive functions in preclinical studies in rodents (Nakazato et al., 2000; Huang et al., 2004). In addition, and in a clinical study, the density of H3Rs in the dorsolateral prefrontal cortex was inversely correlated with performance in the working memory paradigm (Ito et al., 2018).

Contrarily, and in another preclinical study, histaminergic interactions with both H1- and H2Rs were found to be involved in the process of enhancing spatial memory and following i.c.v. administration of HA (Xu et al., 2005). In this study, bilateral ventral intrahippocampal infusion of NMDA receptor antagonist MK-801 impaired the retrieval process in both working memory and reference memory. However, intrahippocampal injection of HA or intraperitoneal injection of histidine markedly ameliorated the spatial memory deficits induced by MK-801. Moreover, both the H1R antagonist pyrilamine and the H2R antagonist cimetidine abolished the ameliorating effect of histidine on reference memory deficits, but not that on working memory deficits induced by MK-801. Accordingly, the latter observations demonstrate that in addition to H1Rs, H2Rs could be involved in the memory processes in experimental animals when memory deficits are induced (Xu et al., 2005). Moreover, physostigmine, an acetylcholinesterase inhibitor, was found to reverse the impairment induced following the administration of an H1R antagonist in working memory, demonstrating the interrelationship of brain histaminergic and cholinergic neurotransmissions in the observed effects on cognitive functions of tested animals (Nakazato et al., 2000). Furthermore, H3-heteroreceptors were extensively reported, as discussed above, to directly influence the release of other neurotransmitters including ACh, glutamate, and NE, all of which are capable of facilitating cognition and attention. Interestingly, atomoxetine and methylphenidate, clinically established drugs in the treatment of ADHD and working memory deficits, were found to increase the release of brain HA in the prefrontal cortex among their pharmacological roles (Horner et al., 2007). Therefore, brain HA may participate in the improvement of cognition and attention necessary for working memory performance.

#### 3.1.3 Long-Term Memory and Brain Histamine

Information intended for long-term learning is further processed. Such processing involves further changes in signaling pathways and consequently altered gene expression for prolonged and larger storage (Mallakin, 2020). STM and LTM are viewed in many studies as separate processes; therefore, it is not uncommon for a particular disease or treatment to affect one of these memory types while sparing the other. The hippocampus is well reported to be the encoding structure for LTM (Passani et al., 2017; Santangelo et al., 2017). Other structures are additionally involved like the basolateral amygdala, septum, cerebellum, and prefrontal cortex (Benetti et al., 2015; Passani et al., 2017; Rani et al., 2021). LTM can be divided into two categories, namely, explicit and implicit memories, which will be discussed in the following in association with the brain HA-provided effects in preclinical studies.

##### 3.1.3.1 Explicit (Declarative) LTM

Conscious and attentive recall of events (episodic) and facts (semantic) is classified as explicit memory (Camina and Güell, 2017). Inhibitory avoidance task that studies fear memory depends on conscious recall of unpleasant, fearful events associated with a specific context (Atucha and Roozendaal, 2015). Histamine H1-, H2-, and H3Rs were reported to be involved in LTM formation. However, differential distribution of HR subtypes in different brain areas influences the contribution of each receptor subtype to the memory process in different behavioral tasks (Figure 1). Accordingly, post-training intra-hippocampal CA1 administration of H3R agonist imetit was found to impair long-term consolidation in object recognition paradigm in rats (da Silveira et al., 2013). Also, this impairment was mimicked following the administration of H1- and H2R antagonists (da Silveira et al., 2013). In addition, HA was reported to regulate memory consolidation and to facilitate the consolidation of extinction (Passani et al., 2017). Furthermore, brain HA was reported to be indeed a major regulator of memory consolidation in various tasks, through H2Rs in the dorsal hippocampus and through H3Rs in the basolateral amygdala, depending on the task (Passani et al., 2017). Accordingly, intrahippocampal administration of HA was found to improve fear memory and was shown to be H2R-dependent when test animals were treated with the H2R antagonist in the pot-training phase, but H1R-dependent in the consolidation phase if given in the pretest phase (retrieval phase) (Passani et al., 2017). Noteworthy, systemic administration of different H3R antagonists/inverse agonists was shown to improve LTM in several studies (Sadek et al., 2016a; Sadek et al., 2016b; Eissa et al., 2018). Also and in numerous other preclinical studies, contradictive results were observed in regard to the pro-cognitive effects of H3R antagonists/inverse agonists, as some were found to be dependent on H1- and/or H2Rs, and memory-enhancing effects of other H3R antagonists/inverse agonists were confirmed to be not dependent on signaling to H1- or H2Rs (Onodera et al., 1998; Sadek et al., 2016a; Alachkar et al., 2017). Furthermore, H3R effects on several memory stages were also reported to be independent of HA release, since H1R-, H2R-, or HDC^-/-^-KO mice or mice with depleted brain HA were reported to be useful experimental rodents for contextual fear memory, and this is in disagreement with the aforementioned observations in numerous preclinical studies on the involvement of the histaminergic system in memory processes (Liu L. et al., 2007; Gong et al., 2010).

##### 3.1.3.1 Implicit (Nondeclarative) Memory

Procedural memory is a type of memory that does not involve consciousness or effort while being recalled (Camina and Güell, 2017). Habits and skills, like driving a car, fall under this category. In preclinical trials, rodents can easily obtain skills in riding rotarods without falling for certain time. Therefore, the time observed prior to falling from rotarods can be utilized to measure implicit memory (Dere et al., 2008). Interestingly, H1R homozygous KO mice were found to display impaired procedural memory in rotarods (Dere et al., 2008). A summary of the preclinical effects observed in association with histaminergic neurotransmission on several memory stages is presented in Figure 1.

#### 3.1.1 Role of H3R Antagonists/Inverse Agonists in Memory Impairment and Cognitive Dysfunction

**FIGURE 2:**
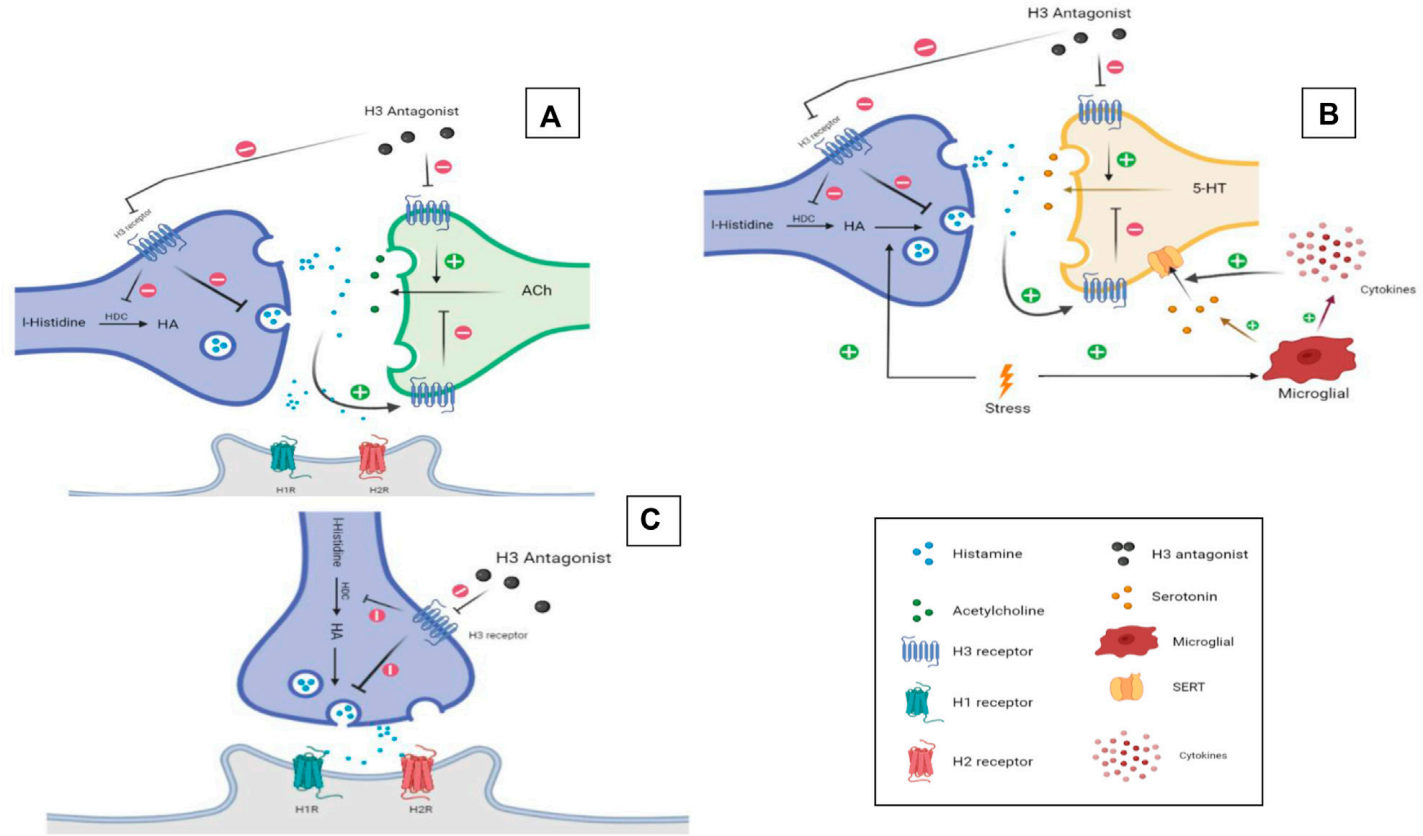
H3R antagonists/inverse agonists. Putative mechanisms underlying memory enhancement effect **(A)**, antidepressant **(B)**, and sleep–wake cycle regulation **(C)** of H3R antagonists/inverse agonists. Graphics were constructed using BioRender software and were licensed for publication.

Numerous H3 antagonists/inverse agonists attracted attention by their capability to alleviate several neurological symptoms in experimental models in rodents. The relationship between HA and memory has been well established in the literature (de Almeida and Izquierdo, 1986; Alvarez, 2009), since H1-, H2-, and H3Rs are expressed in high densities in areas of the brain known to be involved in learning and cognition like the cortex, thalamus, hypothalamus, hippocampus, and amygdala (Martinez-Mir et al., 1990; Pollard et al., 1993). The evidence regarding the involvement of HA receptor subtypes in learning and memory are contradicting. For example, and in an active avoidance task during acquisition, stimulating H1Rs while blocking H2Rs was found to lead to complete inhibition of learning (Alvarez, 2009). Contrarily, stimulating H2Rs while blocking H1Rs yielded a normal learning curve, and the same applies to the retrieval memory phase (Alvarez, 2009). The latter contradictive observations may indicate that H2Rs are solely involved in learning and retrieval processes. However, in a study performed on mice lacking either H1- or H2Rs, both genotypes showed similar learning ability enhancement in the auditory and contextual fear conditioning test, and impairment in the object recognition and Barnes maze task (Dai et al., 2007). In addition, memory enhancement in the passive avoidance paradigm and following systemic administration of several H3R antagonists/inverse agonists has been reported to be moderately reversed by H2R antagonists but not H1R antagonists (Alachkar et al., 2017; Panayi et al., 2017; Eissa et al., 2018). This indicates that H3R antagonist-mediated memory improvement occurs through muscarinic cholinergic receptors and to a lesser extent H2Rs (Leentjens, 2015; Alachkar et al., 2017; Eissa et al., 2018). Overall, the interaction of HA with its receptor subtypes and in the context of memory assessments appears to be task-dependent, complex, and far from being fully understood (Figure 2A). More recently, the role of brain-derived neurotrophic factor (BDNF) has also been suggested (Miranda et al., 2019). Mainly expressed in the hippocampus, cortex, amygdala, and striatum and also in the hypothalamus, BDNF is a protein known to be involved in brain plasticity and depression (Dwivedi, 2009; Miranda et al., 2019). Neuroplasticity is stimulated through neurogenesis, dendritogenesis, and synaptogenesis, and promoting plasticity in dopaminergic, serotoninergic, cholinergic, and noradrenergic neurons. It is also involved in potentiating of signal transmission and induction (Je et al., 2012; Gibon et al., 2016; Liu and Nusslock, 2018). Interestingly, several H3R antagonists/inverse agonists were found to increase brain levels of BDNF in the chronic cerebral hypoperfusion model and age-dependent effect (Guilloux et al., 2017; Wang et al., 2020).

Furthermore, systemic i.c.v. administration of HA in rats was reported to facilitate memory in multiple behavioral models (de Almeida and Izquierdo, 1986; Izquierdo et al., 1992; Prast et al., 1996; Haas and Panula, 2003; Izquierdo et al., 2006; Izquierdo et al., 2016; Sadek et al., 2016a; Sadek and Stark, 2016). The impact of H3R antagonists/inverse agonists on memory can also be mediated by the brain neurotransmitter ACh. As discussed earlier, blocking H3Rs may lead to an increase in brain ACh release, given that ACh depletion in the brain was linked to cognitive impairments in both normal aging and AD patients (Perry et al., 1978; Decker, 1987). Collectively, the evidence showing the effects of anticholinergic agents, e.g., scopolamine and atropine, on memory may indicate another mechanism of enhancing memory impairment by H3R antagonists/inverse agonists (Drachman, 1977; Gallagher and Colombo, 1995). In a recent study evaluating the involvement of the central histaminergic system in social recognition memory on both short and long terms, the H3R antagonist/inverse agonist ciproxifan showed a procognitive effect on LTM in a test of social discrimination. In the same study, the CNS-penetrant H3R agonist was found to impair both short- and long-term social recognition memory (Rani et al., 2021). Interestingly, the genetic or pharmacological blockade of HA release impaired LTM but not STM, and the latter observation may be explained with the actions of H3R agonists on H3-heteroreceptors and the subsequent effect on ACh release. Moreover, Fox et al. reported enhancement of social recognition memory by H3R antagonists/inverse agonists in both adult and aged rats (Fox et al., 2005). Another study explored the effect of H3R antagonist/inverse agonist E159 on memory impairment induced by MK801 using step-through passive avoidance and novel object recognition tasks (Alachkar et al., 2017). The results obtained showed that the H3R antagonist/inverse agonist was able to enhance STM but not LTM through mechanisms related to cholinergic muscarinic neurotransmission and partially by the activation of H2Rs (Alachkar et al., 2017). Accordingly, the results observed for E159 showed that the E159-provided memory-enhancing effects on MK801-induced amnesia were moderately abrogated following acute systemic co-administration of scopolamine, H2R antagonist zolantidine (ZOL), but not with H1R antagonist pyrilamine to the animals (Alachkar et al., 2017). Contrary to the effects observed for E159, and using the same test battery, H3R antagonist/inverse agonist DL77 was found to alleviate deficits of LTM, without any appreciable enhancing effects on STM impairments induced by MK801 in rats (Eissa et al., 2018). The contradictory observations for H3R antagonists/inverse agonists might be explained with the different pharmacokinetic profiles of both compounds, and also probably with the difference in their antagonist affinity to H3Rs with p*K*_i_ of 6.1 and 8.03 for E159 and Dl77, respectively. Also, different species, namely mice and rats, were used in the latter preclinical studies to assess the memory-enhancing effects of both H3R antagonists/inverse agonists E159 and DL77. Furthermore, and in a study that used the 6-OHDA bilateral brain lesion as a model for PD, administration of H3R antagonist/inverse agonist thioperamide ameliorated memory deficits in the novel object recognition task (Masini et al., 2017). Spatial memory is another domain where H3R antagonists/inverse agonists have shown a promising potential therapeutic use. Using Morris water maze—a known behavioral test to assess spatial-working memory, a study showed improvement in scopolamine-induced amnesia following the administration of the H3R antagonist/inverse agonist S 38,093 (Panayi et al., 2017). Likewise, another research group reported improvement in escape latencies and task recall following the systemic administration of the H3R antagonist/inverse agonist GSK189254 in a Morris water maze task and using scopolamine-induced amnesia (Medhurst et al., 2007). Also, the H3R antagonist/inverse agonist ABT-239 enhanced spatial working memory in ketamine-induced memory deficits assessed in a cross maze task (Brown et al., 2013). In this study, ABT-239 and A-431404, but not the reference drugs risperidone and olanzapine, attenuated ketamine-induced deficits on spontaneous alternation in cross-maze. However, both H3R antagonists/inverse agonists failed to affect alternation performance on their own. Moreover, ABT-239 and A-431404 were found to also attenuate MK801-induced impairments in inhibitory avoidance, demonstrating that ABT-239 and A-431404 may have the potential to ameliorate cognitive deficits associated with SCH (Brown et al., 2013). In addition, the H3R antagonist/inverse agonist pitolisant improved episodic-like memory in scopolamine-induced amnesia and natural forgetting situation in male C57BL/6J mice assessed in the two-trial object recognition test (Ligneau et al., 2007). In this study, and on the two-trial object recognition test in mice, a promnesiant effect was observed regarding either scopolamine-induced or natural forgetting, signifying that the H3R antagonist/inverse agonist pitolisant was a valuable drug candidate for further development in wakefulness or memory deficits and other cognitive disorders (Ligneau et al., 2007). Furthermore, the potent H3R antagonist/inverse agonist samelisant, and when combined with sub-effective doses of donepezil, was able to counteract the amnestic effect of scopolamine in the Morris water maze task (Nirogi et al., 2021b). Another study compared the ability of ciproxifan and thioperamide to counteract the amnesic effect of scopolamine in both Morris water maze and Barnes maze; while both were able to diminish the memory impairment in the water maze, only ciproxifan had a less robust effect in the Branes maze (Komater et al., 2005). Also and in another preclinical study, thioperamide was also able to enhance spatial working memory in a cross maze task, although the mice used were not challenged (Vohora et al., 2005). In this study, thioperamide and tacrine enhanced performance on a spontaneous alternation task in mice, and a combination of sub-effective doses of the two drugs had a synergistic effect on the alternation scores in a cross maze test (Vohora et al., 2005). Moreover, sleep-deprived mice developed working memory impairment that was reversed by ciproxifan administration in a T-maze spontaneous alternation task (Chauveau et al., 2014). In addition, sleep restriction failed in the latter study to significantly modify immunopositive cells in control animals; however, ciproxifan administration prevented working memory deficits in sleep-restricted mice through significant increases in Fos labeling in several brain areas, including the prelimbic, infralimbic, and two cingulate cortex regions (Chauveau et al., 2014). Likewise, and in a Y-maze task, the H3R antagonist/inverse agonist SAR110894 reversed cognitive impairment associated with schizophrenia and attention-deficit/hyperactivity disorder, and improved memory performances in several variants of the object recognition task in mice or rats (0.3–1 mg/kg, p.o.) (Griebel et al., 2012). In another behavioral test model, the standard H3R antagonist/inverse agonist pitolisant enhanced consolidation of contextual fear memory and mitigated amnesia induced by dizocilpine (Brabant et al., 2013) (Table 1). In addition, the H3R antagonist/inverse agonist GSK189254 was found to significantly improve performance of rats in diverse cognition paradigms, including inhibitory passive avoidance, water maze, object recognition, and attentional set-shifting, signifying the potential role of targeting H3Rs for the symptomatic treatment of dementia in AD and other cognitive disorders (Medhurst et al., 2007). Furthermore, the H3R antagonists/inverse agonists GT-2331 (Fox et al., 2002) and S38093 (Panayi et al., 2017) were reported to significantly and dose-dependently improve spatial working memory, reverse scopolamine-induced memory deficits, and promote episodic memory of the spontaneously hypertensive rat (SHR) pups in different behavioral paradigms (Table 1). Despite the numerous aforementioned preclinical observations for the effectiveness of several H3R antagonists/inverse agonists to enhance cognitive functions in rodents, a clinical trial revealed that the highly potent and brain penetrant H3R antagonist/inverse agonist GSK239512 used as monotherapy was capable of improving episodic memory in patients with mild-to-moderate AD. However, no improvements were detected for GSK239512 on executive function/working memory or other domains of cognitive functions, indicating that GSK239512 failed to show benefit in this population (Grove et al., 2014). These clinical observations suggested that H3R antagonists/inverse agonists may, at most, have modest and selective effects on cognitive function in patients with mild-to-moderate AD. In addition, another clinical study was carried out with ABT-288, a highly selective H3R antagonist/inverse agonist, demonstrating no efficacy of ABT-288 in the symptomatic treatment of subjects with mild-to-moderate AD (Haig et al., 2014b).

**TABLE 1:** Several H3R antagonists/inverse agonists and their observed effects in preclinical models of memory impairment, depression, anxiety, and sleep–wake cycle disorder.

H3R antagonist/inverse agonist	Dose	Behavioral tests	Animal model	Behavioral outcomes	Reference
A–431404		*Memory*			
	0.3–3.0 mg/kg, i.p.	Cross maze	Ketamine–induced deficit in Male Long–Evans rats	Enhanced spatial working memory	Brown et al. (2013)
	0.3–3.0 mg/kg, i.p.	Inhibitory passive avoidance paradigm	Dizocilpine-induced amnesia in CD1/ICR mice	Enhanced long-term memory retention	Brown et al. (2013)
ABT-239		*Memory*			
	Adult (0.01–0.3 mg/kg) and aged (0.3–1.0 mg/kg) rats	Social recognition	Adult and juvenile Sprague–Dawley rats (24–26 months), and juveniles male Wistar rats	Enhanced recognition memory in both adult and aged rats	Fox et al. (2005)
	1.0–3.0 mg/kg, i.p.	Five-trial inhibitory avoidance	Spontaneously hypertensive (SHR) rat	Improved acquisition of both short- and long-term memory	Fox et al. (2005)
	1.0–3.0 mg/kg, i.p.	Two-choice discrimination water maze	Scopolamine-induced amnesia in adult Long–Evans rats	Partially enhanced spatial memory	Fox et al. (2005)
	0.3–3.0 mg/kg, i.p.	Cross maze	Ketamine-induced amnesia in male Long–Evans rats	Enhanced spatial working memory	Brown et al. (2013)
	0.3–3.0 mg/kg, i.p.	Inhibitory passive avoidance paradigm	Dizocilpine-induced amnesia in CD1/ICR mice	Enhanced long-term memory retention	Brown et al. (2013)
	3 mg/kg, s.c. (21 days)	*Anxiety*	Male Wistar rats	No effect on anxiety-like behaviors	Trofimiuk et al. (2020)
		Elevated plus maze			
Pitolisant		*Memory*			
	15 mg/kg, i.p. (17 days)	Two-trial object recognition	Scopolamine-induced amnesia and natural forgetting situation in male C57BL/6J mice	Enhanced episodic-like memory	Ligneau et al. (2007)
	0.625–20 mg/kg, i.p.	Fear conditioning task	Dizocilpine-induced amnesia in female C57BL/6J mice	Enhanced consolidation and reconsolidation of a contextual fear memory	Brabant et al. (2013)
	20 mg/kg, i.p. for 21 day	*Depression*			
		Forced swim test	Corticosterone-induced depression in male mice CD-1	Failed to ameliorate depression-like behaviors	Kotańska et al. (2020)
Ciproxifan		*Memory*			
	3 mg/kg, i.p.	Social recognition	Histidine decarboxylase gene mice (HDC+/+)	Enhanced short- and long-term recognition memory	Rani et al. (2021)
	3 and 10 mg/kg, i.p.	Two-choice discrimination	Scopolamine-induced amnesia in male Long–Evans rats	Reversed scopolamine-induced amnesia	Komater et al. (2005)
	3 and 10 mg/kg, i.p.	Water maze Barnes circular maze	Scopolamine-induced amnesia in C57Bl/6J mice	Modestly enhanced spatial working memory	Komater et al. (2005)
	3 mg/kg, i.p.	T-maze	Sleep-restricted C57Bl/6J	Enhanced working memory	Chauveau et al. (2014)
	3 mg/kg, i.p. (21 days)	*Depression* Forced swim test	Depression induced by chronic stress in C57Bl/6J mice	Improved depression-like behavior by reducing immobility time	Kumar et al. (2019)
	3 mg/kg, i.p. (21 days)	Tail suspension test	Depression induced by chronic stress in C57Bl/6J mice	Improved depression-like behavior by reducing immobility time	Kumar et al. (2019)
	3 mg/kg, i.p. (21 days)	Social behavior test	Depression induced by chronic stress in C57Bl/6J mice	Improved depression-like behavior by increasing time spent in the social chamber	Kumar et al. (2019)
	3 mg/kg, i.p. (21 days)	Sucrose preference test	Depression induced by chronic stress in C57Bl/6J mice	Reduced anhedonia	Kumar et al. (2019)
	3 mg/kg, i.p. (21 days)	*Anxiety* Elevated plus maze	Male C57Bl/6J mice	No effects on anxiety-like behaviors	Chauveau et al. (2019)
Clobenpropit		*Memory*			
	5 mg/kg, s.c.	Inhibitory passive avoidance paradigm	Male Flinders sensitive line rats	Enhanced memory effect by increasing step through latency time	Femenía et al. (2015)
	5 mg/kg, s.c.	Novel object recognition	Male Flinders sensitive line rats	Enhanced episodic-like memory	Femenía et al. (2015)
	10 mg/kg p.o.	*Depression* Forced swim test	Male Flinders sensitive line rats	Ameliorated depression-like behavior by reducing immobility time	Gao et al. (2013b)
	10 mg/kg p.o.	*Anxiety* Novelty suppressed feeding	Male Flinders sensitive line rats	No effects on anxiety-like behaviors	Gao et al. (2013b)
	5 mg/kg, s.c.	Social interaction	Male Flinders sensitive line rats	No effects on anxiety-like behaviors	Femenía et al. (2015)
	5 mg/kg, s.c.	Light/dark test	Male Flinders sensitive line rats	No effects on anxiety-like behaviors	Femenía et al. (2015)
DL77		*Memory*			
	2.5, 5, and 10 mg/kg, i.p.	Inhibitory passive avoidance paradigm	Dizocilpine-induced amnesia in male Wistar rats	Enhanced memory effect by increasing step through latency time	Eissa et al. (2018)
	2.5, 5, and 10 mg/kg, i.p.	Novel object recognition	Dizocilpine-induced amnesia in male Wistar rats	Enhanced memory effect by modulating exploration time of novel object	Eissa et al. (2018)
	2.5, 5, and 10 mg/kg, i.p.	*Anxiety*	Male Wistar rats	No effects on anxiety-like behaviors	Eissa et al. (2018)
		Elevated plus maze			
E159		*Memory*			
	2.5–10 mg/kg, i.p.	Inhibitory passive avoidance paradigm	Dizocilpine-induced amnesia in male Wistar rats	Enhanced memory effect by increasing step through latency time	Alachkar et al. (2017)
	2.5–10 mg/kg, i.p.	Novel object recognition	Dizocilpine-induced amnesia in male Wistar rats	Enhanced short-term memory by modulating exploration time of novel object. No effects on long-term memory	Alachkar et al. (2017)
	2.5–10 mg/kg, i.p.	*Anxiety*	Male Wistar rats	No effects on anxiety-like behaviors	Alachkar et al. (2017)
		Elevated plus maze			
Enerisant		*Memory*			
	0.03, 0.1 and 0.3 mg/kg, p.o.	Novel object recognition	Scopolamine-induced amnesia in male Wistar rats	Enhanced episodic-like memory	Huang et al. (2006)
	1, 3 and 10 mg/kg, p.o.	*Sleep–Wake Cycle*	EEG sleep–wake regulation in male Sprague–Dawley (SD) rats	Increased wakefulness	Hino et al. (2020)
				Decreased slow-wave sleep	
		*Memory*			
	1 and 3 mg/kg, p.o.	Inhibitory passive avoidance paradigm	Scopolamine-induced amnesia in male Wistar rats	Decreased amnesia in tested rats	Medhurst et al. (2007)
	1 and 3 mg/kg, p.o.	Water maze	Aged male Wistar rats	Decreased platform escape latency	Medhurst et al. (2007)
	0.3 and 1 mg/kg, p.o.	Novel object recognition	Male Lister hooded rats	Increased time spent exploring novel object	Medhurst et al. (2007)
	1 mg/kg p.o.	Object attentional set shift	Male Lister hooded rats	Improved reversal learning	Medhurst et al. (2007)
					Hino et al. (2020)
GT-2331		*Memory*			
	1 mg/kg s.c.	Inhibitory passive avoidance paradigm	Spontaneously hypertensive (SHR) rats	Improved memory effects by increasing step-through latency time	Fox et al. (2002)
JNJ-10181457		*Memory*			
	10 mg/kg, i.p.	Delayed non-matching to position (DNMP) and reversal learning task	Scopolamine-induced deficits and reversal learning task in Sprague–Dawley rats	Increased percentage correct responding in learning	
		*Depression*			
	10 mg/kg, i.p.	Tail suspension test	Lipopolysaccharide (LPS)-induced depression in CX3C chemokine receptor 1 (CX3CR1)-green fluorescent protein (GFP) mice	Improved depression-like behaviors by reducing immobility time	Galici et al. (2009)
	10 mg/kg, i.p.	*Anxiety*	Male C57BL/6 mice, H1RKO and H2R gene knockout (H2RKO) of the C57BL/6 strains	Anxiogenic effects reversed with H2R antagonist	Iida et al. (2017)
	10 mg/kg, i.p.	Elevated zero maze			
		Open field test			
S38093		*Memory*			
	1.1 mg/kg, p.o.	Morris water test	Male Wistar rats	Enhanced spatial working memory	Panayi et al. (2017)
	0.3 and 1 mg/kg p.o.	Novel object recognition	Scopolamine-induced amnesia in male Sprague Dawley rats	Enhanced episodic-like memory	
	0.3 and 1 mg/kg p.o.	Social recognition	Male Wistar rats	Enhanced episodic recognition memory	
Samelisant		*Memory*			
	0.3 to 3 mg/kg, p.o.	Social recognition	Scopolamine-induced amnesia in adult male Wistar rats	Enhanced episodic recognition memory	Nirogi et al. (2021b)
	0.3 to 3 mg/kg, p.o.	Novel object recognition	Scopolamine-induced amnesia in adult male Wistar rats	Enhanced episodic-like memory	Nirogi et al. (2021b)
	0.3 to 3 mg/kg, p.o.	Morris water maze	Scopolamine-induced amnesia in adult male Wistar rats	No effects on spatial working memory as standalone compound, but enhanced working memory when combined with sub-effective dose of donepezil	Nirogi et al. (2021b)
	0.3 to 3 mg/kg, p.o.	Social recognition	Time delay-induced memory defect in adult Wistar rats	No effects on reference memory alone or combined	Nirogi et al. (2021b)
	10 and 30 mg/kg, p.o.	*Sleep–Wake Cycle*	EEG sleep–wake cycle regulation in orexin knock-out mice	Enhanced episodic recognition memory	Nirogi et al. (2021a).
		EEG sleep–wake cycle		Increased wakefulness with a concomitant decrease in NREM sleep	
				Significant decrease in Direct REM sleep onset (DREM) episodes	
Thioperamide		*Memory*			
	20 mg/ kg, i.p.	Novel object recognition	6-Hydroxydopamine (6-OHDA)-induced brain lesion in C57BL/6N	Increased episodic recognition memory	Masini et al. (2017)
	*3 and 10* *mg/kg, i.p.*	Two-choice discrimination water maze	Scopolamine-induced amnesia in male Long–Evans rats	Decreased amnesia	Komater et al. (2005)
	*3 and 10* *mg/kg, i.p.*	Barnes circular maze	Scopolamine-induced amnesia in C57Bl/6J mice	Failed to enhance spatial working memory	Komater et al. (2005)
	7.5 mg/kg, i.p.	Cross maze	Male Swiss Albino mice	Improved spatial working memory	Vohora et al. (2005)
	20 mg/ kg, i.p.	*Depression*	Bilateral partial 6-OHDA lesion in mice	Restored normal rest/activity cycle	Masini et al. (2017)
		HM2 rodent activity monitor system			
ST-1283		*Anxiety*			
	5 mg/kg and 7.5 mg/kg, i.p.	Open field test	Male C57Bl/6J mice	Anxiolytic-like effects	Bahi et al. (2014)
	5 mg/kg and 7.5 mg/kg, i.p.	Elevated plus maze	Male C57Bl/6J mice	Anxiolytic-like effects	Bahi et al. (2014)
	5 mg/kg and 7.5 mg/kg, i.p.	*Depression*	Male C57Bl/6J mice	Improved depression-like behaviors	Bahi et al. (2014)
	5 mg/kg and 7.5 mg/kg, i.p.	Forced swim test	Male C57Bl/6J mice	Improved depression-like behaviors	Bahi et al. (2014)
	5 mg/kg and 7.5 mg/kg, i.p.	Tail suspension test	Male C57Bl/6J mice	Improved depression-like behaviors by reducing feeding latency	Bahi et al. (2014)
		Novelty suppressed feeding test			
SAR110068		*Memory*			
	In rats (0.3–1 mg/kg, p.o.)	Y maze	Male Sprague–Dawley, Wistar	Reversed memory deficits in both rats and mice	Griebel et al. (2012)
	In mice (0.3–3 mg/kg, p.o.)	*Sleep–Wake Cycle*	Female Wistar Han rats and male CD1 mic	Increased wakefulness	
	3 and 10 mg/kg, p.o.	EEG sleep–wake cycle	EEG sleep–wake cycle regulation in male Sprague–Dawley rats	Decreased slow-wave sleep	Gao et al. (2013a)
				Decreased REM sleep	
E100		*Anxiety*			
	5, 10, and 15 mg/kg, i.p. (21 days)	Open field test	Valproic acid-exposed male C57Bl/6J mice	Anxiolytic-like effects	Eissa et al. (2019)
	5, 10, and 15 mg/kg, i.p.	Elevated Plus maze	BTBR T+ tf/J mouse model of autism	Modulated disturbed anxiety levels	Eissa et al. (2020)
		Open field test			

Abbreviations: p, intraperitoneal; p.o., peroral; s.c., subcutaneous.

### 3.2 Anxiety

Anxiety disorders are the most common group of neuropsychiatric disorders in the general population. They are also important because of their association with significant impairment in functioning and with high direct and indirect costs. Anxiety disorders are often associated with depressive disorders and may have other complications (McEnery et al., 2019). A study group analyzed data from the World Mental Health Survey Initiative and found that patients diagnosed with social anxiety disorders (SAD) suffer, also, from impairments in domains such as relationships and social situations as well as key impairments at home and work (Sultzer et al., 1993; Ballard et al., 2000; Aarsland et al., 2001; Porter et al., 2003; Stein et al., 2017). Anxiety symptoms are common among patients with AD. A study aimed to establish defined criteria to diagnose anxiety in patients with dementia and AD, and to explore the prevalence of generalized anxiety disorder (GAD). Accordingly, the observations concluded that 26% of AD patients experienced excessive anxiety and worry in a period of 6 months prior to psychiatric evaluation, experiencing symptoms that include restlessness, irritability, muscle tension, fears, and respiratory symptoms (Starkstein et al., 2007). Furthermore, and based on the same criteria, 10% of AD patients were diagnosed with GAD. Moreover, a pilot study reported greater decline in global cognition, executive functions, and language in AD patients who were also found to suffer from anxiety symptoms (Pietrzak et al., 2015). Therefore, the severity of cognitive decline is linked to anxiety symptoms. Moreover, patients who were diagnosed with AD at an early onset showed higher levels of anxiety that may be attributed to the decline in cognition at earlier stages of life, thus experiencing more difficult challenges and functional disabilities (Kaiser et al., 2014). In addition, anxiety is also thought to be a risk factor of AD (Becker et al., 2018; Santabárbara et al., 2020). In a study of almost 5 years of follow-up, Santabárbara et al. found that clinically relevant anxiety increased the risk of developing AD by four folds (Santabárbara et al., 2019). Moreover, patients with mild cognitive impairment were more likely to develop AD after experiencing anxiety symptoms over a 3-year period (Palmer et al., 2007). A debate of whether such symptoms arise from specific neuropathological changes or are merely a reaction to the cognitive decline in patients is still questionable to researchers. Among the neuropsychiatric profile of PD patients, anxiety is one of the most commonly reported symptoms (Leentjens et al., 2011). In PD patients, symptoms of anxiety are inconsistent among patients and often do not meet any criteria of anxiety subtypes. Such phenomenon is termed as “not-otherwise specified (NOS) anxiety disorder.” NOS anxiety disorder is often followed by specific phobia, panic disorder, and social phobia (Pontone et al., 2009). PD patients with anxiety symptoms are three times more likely to present cognitive dysfunctions, specifically memory impairment, than patients without anxiety (Dissanayaka et al., 2017). Anxiety is not the only symptom that alters the quality of life in PD patients; however, a recent longitudinal study evidenced that social anxiety is highly associated with quality of life measures and social functioning in patients with SCH (Nemoto et al., 2020). Also, cognitive functions such as visuospatial perception, visual memory executive functions, and cognitive flexibility are more commonly impaired in SCH patients with comorbid obsessive compulsive disorder (OCD) and are correlated with its severity (Schirmbeck et al., 2013). Compared with the general population, epilepsy is associated with higher anxiety prevalence, especially in patients with drug-resistant epilepsy suffering from generalized and separation anxiety disorders (Tellez-Zenteno et al., 2007; Scott et al., 2017; Scott et al., 2020). One study group reported the existence of anxiety behaviors in rat models of human epilepsy prior to the onset of seizure, suggesting an overlap underlying pathology of the two disorders (Scott et al., 2017).

#### 3.2.1 Role of H3R Antagonists/Inverse Agonists in Anxiety Behaviors

Brain HA plays an important role in anxiety, as there have been numerous studies indicating a functional relationship between anxiety and histaminergic neurotransmission in classical animal models. Accordingly, the H1R antagonist chlorpheniramine improved anxiety of assessed rats in the elevated plus maze test and the open field test (Hasenohrl et al., 1999). Moreover, several research groups reported different effects of brain HA in preclinical models of anxiety in both male and female mice lacking HDC enzyme (HDC−/−). Accordingly, (HDC−/−)phenotypes were found to present behavioral features related to an increased anxiety level, which are mostly confirmed through animal behaviors in elevated plus maze, zero maze, light dark test (Acevedo et al., 2006), open field test (Dere et al., 2004; Acevedo et al., 2006), and graded anxiety test and height-fear task (Dere et al., 2004). Moreover, mice knocked out of H3Rs showed reduced anxiety in both elevated plus maze and zero maze, but not in the acoustic startle test (Rizk et al., 2004). Furthermore, rats that received bilateral HA infusion in the lateral septum showed decreased anxiety response in novelty-induced suppression of feeding (NISF) test and elevated plus maze (Chee et al., 2014). Intriguingly, the anxiolytic effect observed in the NISF test seems to be mediated by the histaminergic activation of postsynaptic H1R and H2Rs, since administration of either antagonist abrogated the HA-provided effects. However, administration of an H3R antagonist/inverse agonist was reported to reverse the effect seen in elevated plus maze, indicating possible signaling mediated by H3Rs (Chee and Menard, 2013). Conversely, the effect of HA injection on the lateral septal was reported to induce anxiety-like behaviors in the open field test, which was possibly mediated by neurotransmission through H1Rs and H2Rs (Mohsen et al., 2014). The reported results for numerous H3R antagonists/inverse agonists on anxiety models also differed momentously, with the most observed effect being the lack of any effects on anxiety-like behaviors assessed for ABT-239, ciproxifan, clobenpropit, DL77, and E159 in both rats and mice (Femenía et al., 2015; Alachkar et al., 2017; Masini et al., 2017; Eissa et al., 2018; Alachkar et al., 2019; Chauveau et al., 2019; Eissa et al., 2019; Soliani et al., 2020; Trofimiuk et al., 2020). However, the existing studies found that some H3R antagonists/inverse agonists, e.g., the H3R antagonist/inverse agonist ST-1283, may have an anxiolytic effect using paradigms such as open field test and marble-burying test (Bahi et al., 2014; Eissa et al., 2019). On the other hand, the contradicting results indicated an anxiogenic effect of the H3R antagonist/inverse agonist JNJ-10181457 observed in the open field test and the elevated zero maze test, and the anxiety parameters were explained with the increase in the locomotor activity witnessed with this test compound. In addition, the JNJ-10181457-induced anxiogenic and locomotor effects were reversed for the most part by co-administration with an H2R antagonist, suggesting a possible role of the H2Rs in exploratory and anxious behaviors of tested animals (Mohsen et al., 2014). The contradictory observations for the involvement of brain histaminergic neurotransmission in anxiety-like behaviors of experimental rodents shed light on the different task models and animal species used to evaluate the effects of different antagonists targeting H1-, H2-, or H3Rs. Consequently, there is no conclusive statement of the proved anxiolytic and/or anxiogenic effectiveness of several H3R antagonists/inverse agonists.

### 3.3 Depression

Depression is one of the oldest and most recognized medical conditions that affects mood, motor, and neurovegetative functions and cognition (Fava and Kendler, 2000). Patients with major depression disorders (MDD) are recognized to be at high risk for developing cardiovascular disease, diabetes, and dementia (Boulenger et al., 2006; Nouwen et al., 2019; Holmquist et al., 2020), including AD-mediated dementia (Green et al., 2003). The link between severity of depression and the risk of developing dementia has been assessed in a 14-year longitudinal study, and higher risks for developing dementia were found in patients with more severe depressive symptoms (Almeida et al., 2017). Accordingly, a recent guideline by the WHO on risk reduction of cognitive decline and dementia concluded a significant association between depression and dementia (Minghui et al., 2019). Furthermore, depression can lead to a steep decline in cognitive integrity in AD patients (Rapp et al., 2011). A study that compared neuropathological changes in the hippocampus of AD patients, with or without a lifetime history of major depression including neuritic plaques and neurofibrillary tangles, reported that such changes are more profound in patients with a history of major depression (Rapp et al., 2006). Thus, it correlates to a more severe decline in cognitive functions. This association between depression and cognitive decline is also seen in PD patients; a longitudinal study found that elevated baseline depression and anxiety are the two strongest predictors of cognitive decline in domains such as learning. The former research group found no association between these neuropsychiatric symptoms and cognitive impairments in healthy controls, which may suggest a unique association with PD (Pirogovsky-Turk et al., 2017). Depression is common among PD patients, with a prevalence of 39%. As depression is an early prodromal symptom or a risk factor, the nature of such an association is still an area of debate due to the complex pathology of depression (Gustafsson et al., 2015; Leentjens, 2015; Lubomski et al., 2020). Noteworthy, the effect of pain on depression is an area of research that requires further exploring since most PD patients suffer from chronic pain (Mylius et al., 2015). Although depression is long known to be a factor in the prognosis of SCH, it can impair the quality of life independently of negative symptoms and psychosis (Andrianarisoa et al., 2017; McGinty and Upthegrove, 2020). The exact prevalence of depression in SCH is not fully determined as the data in the literature ranged from 16% to 69% (Bressan et al., 2003; Jeyagurunathan et al., 2017). More recently, a major depressive disorder has been reported to be present in approximately one-third of patients diagnosed with SCH (Etchecopar-Etchart et al., 2021). Noteworthy, epilepsy is another neuropsychiatric disorder where depression is a common comorbidity and a factor affecting the progression of the disease. Furthermore, depression was found to be a strong predictor of quality of life and associated with premature mortality (Fazel et al., 2013), with some patients reporting depression to be more disabling than seizures (Boylan et al., 2004). Not only epileptic patients are more likely to suffer from depression than healthy controls (Scott et al., 2017), but they are also at six-fold higher risk to develop seizures (Ortega et al., 2013).

#### 3.3.1 Role of H3R Antagonists/Inverse Agonists in Depression

H3R antagonists/inverse agonists were also reported to have an antidepressant-like effect in experimental rodents (Lamberti et al., 1998; Pérez-García et al., 1999; Bahi et al., 2014; Femenía et al., 2015). However, the exact mechanism of the observed antidepressant-like effects is not fully explored in the literature. A study that explored possible mechanisms of the antidepressant actions of H3R antagonists/inverse agonists addressed the possibility of the involvement of BDNF. This neurotrophic factor is known to be involved in brain plasticity and mood, and recently was linked to depression (Dwivedi, 2009; Miranda et al., 2019). Stress and depression were reported to be capable of reducing the concentrations of BDNF in the hippocampus and prefrontal cortex, as several studies reported restoration of the BDNF factor upon administration of several reference antidepressant drugs (Castrén and Rantamäki, 2010; Yu and Chen, 2010). In preclinical models, mice exposed to chronic unpredictable stress were found to exhibit reduced levels of the BDNF factor in the hippocampus and prefrontal cortex, which was reversed by the administration of ciproxifan, a very well-known standard H3R antagonist/inverse agonist used in numerous preclinical studies in rodents (Kumar et al., 2019). The former researchers also found that the effect of HA on the BDNF factor in primary neurons can be fully blocked using H4R antagonists, indicating a possible role of H4Rs in restoring BDNF levels. This is in correlation with another study that reported depression-like symptoms in H4R-knockout (KO) mice (Sanna et al., 2017). Noteworthy, the HA role in immune response is also integrated in depression pathophysiology. The monoamine hypothesis of major depression relies on the notion that levels of multiple neurotransmitters in the brain are disrupted, specifically 5-HT which is found to be lower in depressive patients (Krishnan and Nestler, 2008; Belmaker and Agam, 2009). Another hypothesis is the possible role of immune response in initiating depression and its progression, and this is mainly through the release of proinflammatory cytokines (Haase and Brown, 2015). HA can be viewed as a link between the two hypotheses by means of its vital role in brain immune responses. Initially, stress can induce HA production either by direct activation of neuronal HA or by increasing the microglia production. Thereafter, HA can lead to decreased production of 5-HT through interaction with H3-heteroreceptors. Furthermore, stress can induce the microglia to produce proinflammatory cytokines that leads to further decrease in 5-HT levels in the synaptic cleft through 5-HT transporters (SERT) (Hersey et al., 2021). In line with the previous findings, a study has found that HA infusion was capable of preventing lipopolysaccharide (LPS)-induced cytokine release and neuronal loss in experimental mice (Saraiva et al., 2019), and H3R antagonism was also found to reduce interleukin (IL)-1β production (Iida et al., 2017). It was also shown that agents such as selective 5-HT reuptake inhibitors and lipid mediator oleoylethanolamide with antidepressant activity required the integrity of the intact brain histaminergic neurotransmission system to exert their effects (Figure 2B) (Munari et al., 2015; Costa et al., 2018). Noteworthy, and in two different studies that tested the effect of the H3R antagonist/inverse agonist ciproxifan on depression induced by chronic unpredicted stress, an elevation of depression-like symptoms such as anhedonia, helplessness, and social deficits was reported in numerous rodent models, including forced swim test, tail suspension test (Je et al., 2012), sucrose preference test, and social behavior test (Kumar et al., 2019), respectively. Furthermore, a study group reported a reversing effect of the H3R antagonist ciproxifan on the BDNF factor (Kumar et al., 2019). The old-generation H3R antagonist/inverse agonist clobenpropit was also found to have a similar effect on depressed rats in a forced swim test, possibly mediated by the actions of released HA on postsynaptically located H1- and H2Rs (Femenía et al., 2015). Moreover, and in the LPS-induced depression model in mice, the H3R antagonist/inverse agonist JNJ-10181457 was found to exhibit antidepressant-like effects by reducing the immobility time of tested mice in the tail suspension test, with evidenced reducing effects on the release of proinflammatory cytokines from microglial cells (Iida et al., 2017). Also, the novel H3R antagonist/inverse agonist ST-1283 was found to reduce depression-like behaviors in forced swim test, tail suspension test, and suppressed feeding test (Bahi et al., 2014) (Table 1).

Noteworthy, a very recent review discussed the association between altered neuroinflammation and brain development, e.g., impacting synaptic plasticity and synaptogenesis, and there were suggestions that HA deficiency may leave the developing brain more vulnerable to proinflammatory insults and neurodevelopmental disorders, including Tourette’s syndrome, autism spectrum disorders, attention-deficit hyperactivity disorder, and SCH, in both preclinical and clinical studies (Carthy and Ellender, 2021)

### 3.4 Sleep–Wake Cycle Disorders

Sleep–wake cycle is regulated by a complex interaction among neurotransmitters and the suprachiasmatic nucleus (SCN) (Siegel, 2004). Dysregulations to this cycle can appear as insomnia, excessive daytime sleepiness, or irregular sleep–wake periods throughout the day (Bjorvatn and Pallesen, 2017). Sleep disturbances are also associated with more severe psychotic episodes, psychosocial impairments (Lunsford-Avery et al., 2017), and poorer health-related quality of life (Batalla-Martín et al., 2020). The pathological mechanisms behind these dysregulations are not fully understood. In addition to genetic factors, loss of ability to generate the circadian rhythm is a proposed mechanism, in addition to loss of neurons in SCN which is a hallmark in neurodegenerative diseases such as AD and PD (Barone et al., 2009; Bjorvatn and Pallesen, 2017; Lunsford-Avery et al., 2017; Batalla-Martín et al., 2020). Sleep disturbances are a common and debilitating complication of AD seen in 25–44% of patients (Vitiello and Borson, 2001). The interplay between abnormal deposition of Aβ protein in the brain of AD and sleep disturbance patients can be viewed as a bidirectional relationship. Recent studies reported compelling evidence in the role of Aβ in the sleep–wake cycle, being present in higher levels in the brain interstitial fluid during wakefulness unlike during sleep time (Kang et al., 2009). Furthermore, sleep latency, quality, and duration were associated with a high Aβ burden (Spira et al., 2013; Brown et al., 2016). Stabilization of memory function and the consolidation process is linked to the sleep cycle, in addition to impairment in synaptic plasticity as a consequence of sleep deprivation (Graves et al., 2003; Prince et al., 2014), collectively supporting the hypothesis that sleep disturbances can be a risk factor and an early predictor of cognitive decline. On the other hand, evidence indicating that AD pathophysiology itself can lead to sleep disturbances are also present. Eliminating Aβ plaques from APP/PS1 mice normalized the sleep–wake cycle (Roh et al., 2012), and the presence of Aβ plaques in certain neuronal circuits was attributed to regulating the sleep–wake cycle (Busche et al., 2015). Therefore, disturbances in these circuits are also a proposed mechanism behind sleep dysregulations. Insomnia, excessive day time sleepiness and sleep fragmentation are the most commonly reported complaints among AD patients (Bachman and Rabins, 2006; Guarnieri et al., 2012). Similar disturbances are also witnessed in PD patients; however, the etiology behind these disturbances was described to be of multifactorial nature. Unlike some symptoms such as insomnia, other research groups reported sleep disorders including rapid eye movement, day-time alertness, restless legs syndrome (RLS), periodic limb movement disorder (PLMD), and circadian rhythm dysfunction, and associated these disorders with the neurodegenerative processes and dysregulation in DA, a brain neurotransmitter that plays a vital role in the sleep–wake cycle (Dzirasa et al., 2006; Videnovic and Golombek, 2013). Similar sleep disturbances are also common in SCH, recognizing insomnia and nightmare disorders as the most common, in addition to others such as RLS, PLMD, and circadian dysfunctions (Monti et al., 2013). Given the pathophysiological background of SCH, insomnia can be attributed to abnormal levels of DA (Monti et al., 2013). Sleep disturbances are also of high prevalence in epileptic patients and are likely related to worsened disease outcomes. Moreover, previous preclinical observations in C57B/6J mice indicated that DA regulates the generation of sleep–wake states, proposing that psychosis and the sleep disturbances experienced by PD patients result from DA-mediated disturbances of REM sleep (Dzirasa et al., 2006). Moreover, adults diagnosed with epilepsy are more likely to suffer from sleep disorders than the general population, with insomnia and RLS as the most common symptoms. Lower sleep quality and excessive day-time sleep were found to exacerbate seizure incidences even with appropriate pharmacological control in juvenile myoclonic epilepsy (Buratti et al., 2018). These disturbances can be a consequence of either seizures or antiepileptic drugs or a combination of the two factors (Joutsa et al., 2017). However, recent attention has been paid to the involvement of Aβ pathology in epilepsy and its relation to the reported sleep disturbances, and Aβ has been found to possess the ability to induce epilepsy in animal models and has been linked to epileptic seizures (Liguori et al., 2021).

#### 3.4.1 Role of H3R Antagonists/Inverse Agonists in Sleep–Wake Cycle Disorders

The original basis behind ritualizing the use of H3R antagonists/inverse agonists to treat sleep-related disorders comes from multiple evidence found in the literature. The role of HA in wakefulness is not initiating but more specifically maintaining the state of alertness needed for higher brain functions (Takahashi et al., 2006). Histaminergic neurons project to different areas of the brain that are involved in the sleep–wake cycle such as the cortex, thalamus, hypothalamus, and brain stem (Vanni-Mercier et al., 2003). Brain histaminergic and orexinergic neurons found in the posterior hypothalamus were also found to play a significant role in the sleep–wake cycle. Histaminergic neurons within the tuberomammillary nucleus (TM) and the posterior hypothalamus are marked as “waking selective,” which means that they only fire during the waking state. In fact, these neurons possess the most selective pattern of discharge to wakening status within the CNS (Figure 2C) (Vanni-Mercier et al., 2003). In mice phenotype lacking HDC, decreased sleep latencies were observed. In new surroundings, EEG readings in wild-type mice show significant changes such as increased paradoxical sleep that tends to be preceded with wakefulness for hours, unlike HDC-/- mice that fall asleep spontaneously in comparison with wild-type mice. Furthermore, injecting wild-type mice with the HDC inhibitor α-FMH elicited similar results, thus indicating the importance of brain HA in maintaining the wake state (Parmentier et al., 2002). Likewise, a recent study carried out in mice with reduced expression of HDC and cKO mice using adeno-associated viruses to exclude the chance of developing compensating mechanisms to maintain the sleep–wake cycle concluded that chronic HA depletion resulted in a significant decrease in wakefulness and an increase in nonrapid eye movement (NREM) sleep (Yamada et al., 2020). Also, H1R KO mice and mice administered with an H1R antagonist showed similar disturbances in sleep patterns compared with wild-type mice, except for increased NREM sleep and decreased latencies to initiate NREM (Scott Bitner, 2012). On the other hand, H3R KO mice showed more awakening during changes in environment and motivation tests. Also, H1R antagonists were reported to be precipitating greater in slow-wave sleep in H3R KO mice, supporting the involvement of histaminergic interaction with H1Rs in sleep regulations (Scott Bitner, 2012). Furthermore, numerous H3R antagonists/inverse agonists were studied for their possible therapeutic potential in regulating sleep, some of which, e.g., pitolisant, were approved for treatment of narcolepsy (excessive day-time sleepiness) (Mohsen et al., 2014). Application of H3R antagonists/inverse agonists in preclinical models of sleep disturbances showed great potentials. For instance, GSK189254 enhanced narcoleptic episodes in orexin KO mice; EEG and EMG showed increased wakefulness (W) and decreased paradoxical and slow-wave sleep (Guo et al., 2009). Using the same mouse model, samelisant decreased NREM sleep and direct REM sleep onset episodes, showing an anti-cataplectic effect (Nirogi et al., 2021a). Another H3R antagonist/inverse agonist, namely, enerisant, was able to promote a waking effect by decreasing slow-wave sleep, however, at high doses (Hino et al., 2020). Moreover, the H3R antagonist/inverse agonist SAR110068 produced wakefulness and decreased slow-wave and REM sleep to similar degrees as ciproxifan but for longer durations as shown by EEG (Gao et al., 2013a). In another study, a bilateral lesion of the striatum in mice using 6-OHDA produced a disruption in the normal endogenous circadian rhythm, which was reversed following systemic administration with the H3R antagonist/inverse agonist thioperamide (Masini et al., 2017) (Table 1). Apart from increasing the levels of brain HA, samelisant was also found to modulate DA and NE levels in the cerebral cortex, while it had no effects on DA levels in the striatum or nucleus accumbens (Nirogi et al., 2021a). In addition, systemic treatment with samelisant was reported to produce a significant increase in wakefulness with a concomitant decrease in NREM sleep and direct REM sleep onset (DREM) episodes in orexin knockout mice subjected to sleep EEG, demonstrating its anticataplectic effects in an animal model relevant to narcolepsy (Nirogi et al., 2021a). Importantly, some positive effects of H3R antagonists/inverse agonists have been reported for the narcoleptic rodent model and for patients with narcolepsy (Romigi et al., 2018; Davis et al., 2021; Fabara et al., 2021). Accordingly, pitolisant (BF2.649/tiprolisant/Wakix), one of the H_3_R antagonists/inverse agonists, was reported to enhance HA neuronal activity, promote wakefulness, and decrease abnormal onset of REM sleep from the wakefulness in Hcrt knockout mice (Romigi et al., 2018; Davis et al., 2021). The drug (Wakix®), a first-in-class antagonist/inverse agonist of the H3Rs, was approved in the EU (as of March 2016) for the treatment of narcolepsy with or without cataplexy in adults and in the United States (as of August 2019) for the treatment of excessive daytime sleepiness (EDS) in adults with narcolepsy. Moreover, in patients with narcolepsy, two small trials exhibited the effect of pitolisant on recovery from excessive daytime sleepiness (Romigi et al., 2018; Davis et al., 2021). In addition, pitolisant was reported to ameliorate excessive daytime sleepiness with comparable effectiveness to that of modafinil, an approved medicine for narcolepsy (Romigi et al., 2018; Davis et al., 2021). Therefore, pitolisant is currently assessed in different ongoing or completed clinical trials (clinicaltrials.gov), which may provide more insight into the potential role of brain HA in the excessive sleepiness and cataplexy attacks in narcolepsy (Shan et al., 2015; Carthy and Ellender, 2021; Davis et al., 2021; Fabara et al., 2021).

## 4 Implication of the Histaminergic System in Other Brain Disorders

The correlation between brain HA and neurological diseases is not limited. Aside from the aforementioned disorders such as AD, SCH, and PD, other neurodegenerative diseases such as Huntington’s and neurotrophic scleroses were also reported to be affected by HA regulation (Haas and Panula, 2003; Shan et al., 2015; Sadek et al., 2016a). Huntington’s disease (HD) is a rare progressive neurodegenerative neuropathy that is clinically pictured by chorea dystonia and cognitive impairments (Alachkar et al., 2019), in addition to other less frequent symptoms including circadian rhythm disturbances and weight loss (Soliani et al., 2020). Histaminergic signaling was found to be higher than normal in the HD brain; this may be associated with non-motor symptoms presented such as cognitive impairments, sleep dysregulation, and weight loss (Masini et al., 2017). Symptomatic relief is the only available option for HD patients. However, promising studies enlist H3R antagonists as a potential therapeutic benefit. The use of H3R antagonists, specifically thioperamide, prevented spatial, working, and long-term memory defects in an animal model of HD (Moreno-Delgado et al., 2020). Moreover, the use of GSK189254 in an animal model of HD improved several behavioral aspects including cognitive impairments, sleep–wake cycle dysregulations, and mood (Whittaker et al., 2017). Amyotrophic lateral sclerosis (ALS) is a disease characterized by progressive degeneration of lower and upper motor neurons resulting in a wide range of symptoms including muscle stiffness, spasticity, twitching, and atrophy. However, one-third of the cases are “Bulbar” since they present difficulty chewing, swallowing, and speaking (Brown and Al-Chalabi, 2017). Many HA-related genes were reported to be dysregulated in ALS patients. Administration of the HA precursor, namely histidine, in SOD1-G93A mice that present a model of ALS showed positive effects on the behavioral and neuropathological symptoms, as well as in attenuating disease progression and improving motor functions (Apolloni et al., 2019). Moreover, polymorphism of the Thr105Ile allele precipitates a 60% reduction in HNMT activity and subsequently delays the onset of ALS symptoms in patients (Chen et al., 2018; Volonté et al., 2019). Furthermore, HA has been reported to restore the inflammatory balance in SOD1-G93A mice by modulating pro-inflammatory and anti-inflammatory marker expression. Thus, increasing brain levels of HA can serve as a candidate therapeutic target. Another neuropathy linked to HA modulation is multiple sclerosis (MS), which is a demyelinating inflammatory disease targeting the CNS causing sensory, motor, autonomic, and cognitive impairments as well as axonal loss (Sospedra and Martin, 2004). It was found that experimental autoimmune encephalomyelitis (EAE), the experimental model of MS, is worsened in mice lacking the HDC gene, therefore accounting HA in regulating the immune response against myelin in the EAE model. Myelination can be induced by promoting the development of oligodendrocyte progenitor cells (OPCs) into oligodendrocytes (Kuhlmann et al., 2008); such a mechanism was found to be mediated by different means: first, through HA-induced OPC translocation to sites of inflammation, then by promotion of OPC development into mature oligodendrocytes through H3Rs expressed in neurons and OPCs (Chen et al., 2017), and finally by stimulation of myelin formation (Wright, 2000). GSK239512 (Schwartzbach et al., 2017) and GSK247246 (Chen et al., 2017; Rangon et al., 2018) are both H3R antagonists/inverse agonists that were found to increase the remyelination process in MS patients. Neuropathic pain is best defined as injury or lesions or a disease in the somatosensory system that causes pain (Colloca et al., 2017). The mechanisms behind neuropathic pain are not fully understood. HA association with pain is widely present in the literature and was highly augmented after reporting the presence of H3Rs in nociceptive pathways, indicating its involvement in the regulation of nociceptive transmission (Colloca et al., 2017). Several studies confirmed the former relation using H3R antagonists/inverse agonists on constriction-injury mice as a preclinical pain model. For instance, E162, an example of H3R antagonist/inverse agonist, showed analgesic effects when compared with morphine (Popiolek-Barczyk et al., 2018). However, H1R blockade reduced this effect, confirming its role in this analgesic outcome. Nevertheless, E162 also enhanced the antinociceptive actions of morphine when administered together (Popiolek-Barczyk et al., 2018). Another example is the systemic administration of the H3R antagonist/inverse agonist GSK189254 that potently produced an antinociceptive effect in monoiodoacetate-induced osteoarthritic pain in comparison with celecoxib (Hsieh et al., 2010). Likewise, the H3R antagonist/inverse agonist S38093 was able to elicit a nociceptive effect in different models of pain (traumatic, diabetic, and chemotherapy-induced pain) (Chaumette et al., 2018).

## 5 Conclusion

More than three decades have passed since the role of brain HA in the regulation of several memory stages was first proposed by De Almeida and Izquierdo in 1986 (de Almeida and Izquierdo, 1986), and since then, significant progress has been made, and today, there is compelling evidence that alterations in the brain histaminergic system are linked with the cognitive impairments observed in several neurodegenerative disorders. Since clinical manifestations can coexist in a single patient and, therefore, demonstrate an overlapping and sometimes contradicting pathophysiological basis, the pharmacological intervention against all these comorbid symptoms may be hindered and can make it even more difficult. Consequently, a compelling urge to find a therapeutic agent that can target several of these comorbid impairments is rising. The positive effects of numerous H3R antagonists/inverse agonists in cognitive impairment, AD, PD, SCH, depression, anxiety, and sleep disorders are present in the literature and establish a promising stepping stone to address neuropathological features commonly occurring together. The exclusivity of the function of H3R antagonists/inverse agonists comes from its location as a presynaptic H3-autoreceptor in the CNS, controlling the release of HA, and as H3-heteroreceptor modulating the release of several other critical neurotransmitters such as ACh, GABA, glutamate, NE, 5-HT, and DA. Despite this, the vast range of applications for these agents is immensely neglected and not well implemented in therapeutic approaches. This may be due to the lack of evidence-based behavioral research that may give a better understanding to the mechanisms behind its actions. The contradicting results seen in the effects of different H3R antagonists/inverse agonists on short- and long-term memory deficits as well as on anxiety in rodents can be cleared with a better understanding of the molecular basis of these observed effects. Also, the discrepancies observed within preclinical data and also in comparison with the results of some clinical trials, especially in patients diagnosed with AD and SCH, may inspire the development of new therapeutic strategies for human diseases, and initiate several questions that need to be addressed in future studies using cutting-edge technologies. In addition, it should be mentioned that numerous H3R antagonists/inverse agonists are contemplated as constituents of multifunctional drugs for the treatment of neurodegenerative diseases (e.g., as an active element of dual- or multi-targeting drugs).
